# Supplementary material for: Beyond biomarkers: the role of clinical factors associated with biologic therapy response in severe asthma
Source: Ann Med. 2026 Feb 11;58(1):2627026. doi: 10.1080/07853890.2026.2627026 (PMC12895875; doi:10.1080/07853890.2026.2627026)
Supplement: Revised manuscript clean copy4.docx [file IANN_A_2627026_SM6896.docx]

TITLE:

Beyond Biomarkers: The Role of Clinical Factors Associated with Biologic Therapy Response in Severe Asthma

BRIEF TITLE:

Asthma Biologics: Key Associated Clinical Factors

AUTHORS

Raquel Lopez-Rodríguez^1^, José Antonio Cañas ^2,3^, Ignacio Mahíllo-Fernández ^2,4,5^, Manuel Jorge Rial ^3,6^

^1.^ Allergy Department. Hospital Universitario Lucus Augusti, Lugo, Spain

^2^. Immunology Department, Health Research Institute-Fundación Jiménez Díaz University Hospital, Universidad Autónoma de Madrid (IIS-FJD, UAM), 28040 Madrid, Spain

^3.^ CIBER de Enfermedades Respiratorias (CIBERES), Madrid, Spain

^4.^ Biostatistics and Epidemiology, Fundación Jiménez Díaz, Madrid, Spain^.^

^5.^ Department of Medicine, Universidad Autónoma de Madrid. CIBERES, Instituto Carlos III, Madrid, Spain

^6.^ Allergy Department. Complejo Hospitalario Universitario de Ferrol, Ferrol, Spain

CORRESPONDING AUTHOR: Raquel López-Rodríguez. Allergy Department. Hospital Universitario Lucus Augusti, [Dr. Ulises Romero, 1, 27003, Lugo](https://www.bing.com/ck/a?!&&p=bc39a526ef5d7f3d0c576b26a7620139f4f7f5b885e5e6f476da5af9362d7be2JmltdHM9MTc0MTkxMDQwMA&ptn=3&ver=2&hsh=4&fclid=3ca730dd-a611-6b46-273a-2450a7cc6ad6&u=a1L21hcHM_Jm1lcGk9MTA5fn5Ub3BPZlBhZ2V-QWRkcmVzc19MaW5rJnR5PTE4JnE9SG9zcGl0YWwlMjBVbml2ZXJzaXRhcmlvJTIwTHVjdXMlMjBBdWd1c3RpJnNzPXlwaWQuWU42MzA2eDE4MjI2NDEzMzkyNTQzMDc0MzU0JnBwb2lzPTQzLjAyMDAzODYwNDczNjMzXy03LjUzNTMyNTUyNzE5MTE2Ml9Ib3NwaXRhbCUyMFVuaXZlcnNpdGFyaW8lMjBMdWN1cyUyMEF1Z3VzdGlfWU42MzA2eDE4MjI2NDEzMzkyNTQzMDc0MzU0fiZjcD00My4wMjAwMzl-LTcuNTM1MzI2JnY9MiZzVj0xJkZPUk09TVBTUlBM&ntb=1)  (Lugo), Spain. Rlopezr26@gmail.com

Words: 3916

SUMMARY

Background: Severe asthma is a chronic condition with a high disease burden, often requiring biologic therapy targeting Type 2 (T2) inflammation. However, not all patients achieve optimal control, and traditional biomarkers, such as **blood eosinophils, fractional exhaled nitric oxide (FeNO), and total serum IgE**, may not fully explain variability in treatment response. Identifying additional clinical factors associated with therapeutic success is crucial for optimizing management.

Objective: To evaluate clinical and inflammatory characteristics associated with response to biologic therapies in a real-world cohort of severe asthma patients.

Methods: A single-center, ambispective observational study was conducted in the Allergology Department of A Coruña, Spain. Sixty-seven patients with severe uncontrolled asthma and treated with omalizumab, mepolizumab, benralizumab, dupilumab, or tezepelumab, were included. Patients were followed for at least 12 months, with clinical and inflammatory biomarkers assessed at baseline, 4-6 months, and 12 months. Statistical analyses included comparisons between biologic subgroups and logistic regression models to identify clinical and inflammatory variables associated with treatment response.

Results: Female sex, nasal polyposis, elevated blood eosinophils, and frequent exacerbations were associated with better response to biologics. Clinical factors such as nasal polyposis and aspirin-exacerbated respiratory disease (AERD) showed a stronger association with treatment response than standard biomarkers (FeNO, blood eosinophils). A mean diagnostic delay of 12.1 years was observed, suggesting that diagnostic delay may be associated with differences in treatment outcomes.

Conclusion: Beyond traditional biomarkers, clinical factors such as nasal polyposis and AERD may play a key role in understanding **response patterns** to biologics. Incorporating these variables into clinical assessment may help support a more personalized management approach in severe asthma.

KEY WORDS: Severe Asthma, Biological Therapy, Nasal Polyps, Aspirin-Induced Asthma (Aspirin-Exacerbated Respiratory Disease - AERD), Eosinophilia, Biomarkers, Clinical factors, Precision Medicine

| What do we know about this topic? | Biologic therapies improve severe asthma control, but response varies. Traditional biomarkers (FeNO, eosinophils, IgE) guide treatment selection, yet some clinical factors, like nasal polyposis and AERD, might help explain variability in therapeutic outcomes. |
| --- | --- |
| How does this study impact our current understanding and/or clinical management? | This study suggests that beyond biomarkers, clinical factors such as nasal polyposis and AERD are key factors associated with response of biologic therapy. Incorporating these factors into treatment algorithms could enhance personalized management of severe asthma |

ABBREVIATIONS

1. ABPA – Allergic Bronchopulmonary Aspergillosis
2. ACT – Asthma Control Test
3. AERD – Aspirin-Exacerbated Respiratory Disease
4. ATS – American Thoracic Society
5. BMI – Body Mass Index (Índice de Masa Corporal)
6. CI – Confidence Interval
7. COPD – Chronic Obstructive Pulmonary Disease
8. CRSwNP – Chronic Rhinosinusitis with Nasal Polyps
9. ERS – European Respiratory Society
10. EXACTO – Exacerbations, Asthma Control, Corticosteroids, Treatment Outcomes
11. FeNO – Fractional Exhaled Nitric Oxide
12. FEOS – FEV1, Exacerbations, Oral Steroids, Symptoms score
13. FEV1 – Forced Expiratory Volume in 1 Second
14. FVC – Forced Vital Capacity
15. GEMA – Spanish Guide for Asthma Management
16. ICS – Inhaled Corticosteroids
17. IgE – Immunoglobulin E
18. IL – Interleukin (usado en IL-4, IL-5, IL-13)
19. LABA – Long-Acting β₂-Agonists
20. LAMA – Long-Acting Muscarinic Antagonists
21. LTRA – Leukotriene Receptor Antagonists
22. MEGA – Mechanisms involved in the Genesis and evolution of Asthma Project
23. OR – Odds Ratio
24. PFT-- Pulmonary function Test
25. PSQ-- Psychiatric follow-up
26. RCT – Randomized Controlled Trial
27. SCU—Spontaneus chronic urticaria
28. SD – Standard Deviation
29. SNOT – Sino-Nasal Outcome Test (SNOT-22)
30. T2 – Type 2 (inflammation)
31. TSLP – Thymic Stromal Lymphopoietin
32. VAS – Visual Analog Scale

1. Introduction

Asthma is a highly prevalent chronic disease worldwide, with a significant impact on patients’ quality of life and on the healthcare system. It is estimated that 5-10% of people with asthma present with a severe form of the disease, characterized by a lack of clinical control despite the use of conventional high-dose therapies, such as inhaled corticosteroids (ICS) combined with long-acting β2-agonists (LABA) and long-acting anticholinergics (LAMA) [1]. This population of patients with severe asthma experiences frequent exacerbations, functional limitations and an increased need for medical resources, underscoring the need for more targeted and effective therapeutic options [1,2].

In recent years, the development of biologic therapies has transformed the treatment of severe asthma. These drugs, designed to intervene in specific targets of type 2 (T2) inflammation, have demonstrated efficacy in controlling symptoms and reducing exacerbations. The main biologic agents currently in use include omalizumab, an anti-IgE monoclonal antibody; mepolizumab and reslizumab, which inhibit IL-5; benralizumab, which induces eosinophil apoptosis through its action on the IL-5 receptor (α subunit); dupilumab, an IL-4 and IL-13 receptor antagonist; and, more recently, tezepelumab, which blocks the activity of thymic stromal lymphopoietin (TSLP), a key cytokine in the initial activation of T2 inflammation [1-3]. Unlike other biologics, tezepelumab has also demonstrated clinical benefits in patients with non–T2 asthma phenotypes, reflecting its broader anti-inflammatory mechanism.

However, the clinical characteristics of patients receiving each of these drugs and their therapeutic response vary significantly. Factors such as asthma phenotype, blood eosinophilia level, presence of allergies and other comorbidities often influence the choice of biologic therapy and its clinical efficacy [2-4]. The lack of studies comparing patient characteristics according to the type of biologic used limits the optimization of disease management and individualization of treatments.

In addition, a subgroup of patients with severe asthma has been observed to show an extraordinary therapeutic response to these biologic therapies, known as “super-responders” [5]. These patients experience marked clinical improvement, with an almost complete reduction in exacerbations, greater stability in symptom control and a significant decrease in the use of systemic corticosteroids [2,5]. The identification and characterization of super responders is of great clinical interest, as it could provide valuable information to better characterize response to different biologics and optimize treatment for each patient [5].

Recent real-world evidence further supports the clinical heterogeneity in response to biologic therapies. The Belgian Severe Asthma Registry study demonstrated that patients with chronic rhinosinusitis and nasal polyps showed higher response rates to anti–IL-5 and anti–IL-4/13 biologics compared with those without nasal polyposis, emphasizing the importance of upper airway comorbidities as clinical factors associated with treatment response [6]. Similarly, Valverde-Monge et al. compared long-term remission across omalizumab and anti–IL-5/IL-5R agents, showing that remission can be achieved with all biologics but varies depending on clinical phenotype and response criteria applied [7]. Moreover, a recent scoping review by Pérez-de Llano et al. highlighted the potential benefits of early initiation of biologic therapy, suggesting that delayed treatment introduction may be **associated with reduced long-term effectiveness** [8]. In this context, our ambispective real-world cohort adds value by integrating both retrospective and prospective data to explore how clinical factors—beyond classical biomarkers—can guide therapeutic decisions and are associated with biologic response in severe asthma.

In order to identify clinical patterns associated with each biologic, as well as to evaluate the specific characteristics of the super-responders in each group, we analyzed a cohort of patients with severe asthma treated in the area of A Coruña, Spain, categorizing them according to the biologic drug they received (omalizumab, mepolizumab, benralizumab, dupilumab, reslizumab or tezepelumab). The demographic, clinical, and inflammatory biomarker characteristics of the patients have been analyzed. This analysis aims to contribute to a better understanding of the factors that influence the selection of biologic therapies for severe asthma, to define patient profiles associated with optimized therapeutic response, and to improve the personalized management of this complex disease.

METHODS

*Design and participants*

Single-center, ambispective, observational and real study in the Department of Allergology of Hospital Universitario de A Coruña (Spain). We included 67 patients aged ≥18 years with a diagnosis of severe uncontrolled asthma with biologic treatment (dupilumab, omalizumab, reslizumab, benralizumab, mepolizumab and/or tezepelumab) according to the criteria of the GEMA 5.4 guidelines (Spanish Guideline on Asthma Management) [9] maintained for at least 6 months and with a follow-up of at least 12 months after starting treatment.

All patients who were prescribed biologic therapy for severe asthma in the Allergy Department of Hospital Universitario de A Coruña were consecutively included through the hospital’s official registry up to December 2023. The study began in September 2023; therefore, data from patients who had already initiated biologic therapy before that date were collected prospectively following a standardized protocol. After December 2023, additional prospective data corresponding to visit 1 (5–6 months) and visit 2 (12 months) were recorded to complete follow-up. Only patients who completed at least the first follow-up visit (5–6 months) were eligible for inclusion. Those who discontinued treatment before this point, due to a switch to another biologic, lack of adherence, or adverse events, were excluded during the screening phase and are therefore not represented in the flowchart. A total of 70 patients met inclusion criteria and were analyzed. Subsequently, three patients treated with reslizumab were excluded from the comparative analysis due to the small sample size and lack of comparability with the other biologics, resulting in a final cohort of 67 patients included in the main analysis (Figure 1).


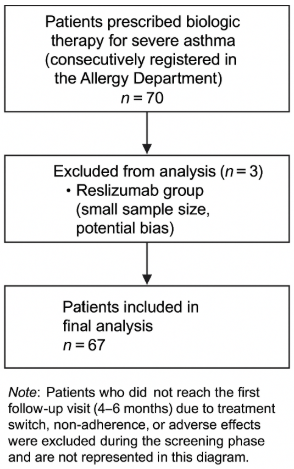


**Figure 1. Flow diagram showing patient selection and exclusion process for the final analysis of severe asthma patients treated with biologics**

*Process*

A retrospective review of electronic medical records was performed for this study. We evaluated outcomes in all patients with severe asthma under continuous treatment with biologics in our Severe Asthma Specialized Unit until December 2023. Data were recorded at three time points: baseline (visit 0) before starting treatment with biologics, at 4-6 months after starting biologic treatment (visit 1), and at 12 months (visit 2). Study variables included demographics, asthma-related comorbidities, atopy (defined by a positive skin prick test to common aeroallergens or by specific IgE assays (ImmunoCAP) when skin testing was contraindicated or not feasible), aspirin-exacerbated respiratory disease (AERD) was diagnosed according to ERS/ATS criteria based on the presence of asthma and/or chronic rhinosinusitis with nasal polyps, together with respiratory symptoms induced by aspirin or other NSAIDs. Chronic rhinosinusitis with nasal polyps (CRSwNP) was diagnosed by clinical symptoms (nasal obstruction, hyposmia, rhinorrhea) confirmed by nasal endoscopy and/or sinus computed tomography (CT) imaging, cardiovascular risk factors, asthma characteristics, whether they were bio-naïve or had switched to another biologic agent, total serum IgE level, peripheral blood eosinophil count, exacerbations, the Asthma Control Test (ACT) score [10] (an ACT score ≥20 indicates well-controlled asthma), lung function, fractional exhaled nitric oxide (FeNO), and systemic corticosteroid use.

Clinical response was evaluated using two validated multidimensional tools: the FEOS score (FEV₁, Exacerbations, Oral corticosteroids, and Symptoms), which ranges from 0 to 100 and reflects overall clinical improvement, and the EXACTO score (Exacerbations, Asthma Control, Corticosteroids, and Treatment Outcomes), ranging from 0–7 (without corticosteroids) or 0–10 (with corticosteroids). Both scales were applied according to their original publications [11,12], with predefined categories to stratify the response as partial, good, or complete.

Exacerbation was defined according to ATS/ERS criteria [13] as acute or subacute episodes of progressive shortness of breath, cough, wheezing and chest tightness, or some combination of these symptoms requiring the use of systemic corticosteroids (tablets, suspension or injection), or an increase in the stable maintenance dose, for at least 3 days or a hospitalization or emergency department visit due to asthma, requiring systemic corticosteroids. Clinical remission was defined as the absence of exacerbations and oral corticosteroid use, with controlled symptoms during follow-up, whereas complete remission additionally required normalization of lung function and inflammatory biomarkers, according to the Menzies-Gow criteria [14,15].

*Statistical analysis:*

Continuous variables were expressed as mean ± standard deviation (SD). Qualitative variables were summarized using frequencies and percentages. Normality of continuous variables was assessed using the Kolmogorov-Smirov test for sample sizes greater than 50, and Shapiro-Wilk test for smaller samples. Frequencies of patients with comorbidities before biologic use were compared between the different biologic subgroups and associations with improvement in those super-responder patients, applying the Chi-square test or Fisher's exact test. Categorical variables were described as absolute frequencies and percentages. For paired samples of continuous data, comparisons were performed using the two-tailed paired samples t-test or the Wilcoxon signed rank test depending on the distribution of the data. Paired comparisons of clinical and inflammatory variables before and after treatment were performed using the Wilcoxon signed-rank test. Comparisons between independent groups, such as different biologic therapies at baseline or follow-up visits, were conducted using the Mann–Whitney U test for two-group comparisons or the Kruskal–Wallis test for three or more groups.Univariable logistic regression models were used to assess the association between clinical and inflammatory variables and treatment response (clinical remission and non-response) at 6 and 12 months. Due to sample size limitations and event distribution, multivariable models could not be constructed. Results are presented as odds ratios (OR) with 95% confidence intervals (CI) and p-values. [14,15]. A p < 0.05 was considered significant. Statistical analyses will be performed with R Foundation for Statistical Computing, Vienna, Austria; [2022]. Models were adjusted for age, sex, and clinically relevant comorbidities (nasal polyposis, AERD). Given the exploratory design and sample size, no formal correction for multiple comparisons was applied, which has been discussed as a study limitation. Given the exploratory and observational nature of the study, these findings should be interpreted as associations rather than as validated predictors of treatment response, and further prospective studies with external validation are warranted.

*Ethical Approval and Informed Consent*

This study received ethical approval from the Comité de Ética de la Investigación (CEIm-FJD) at the Fundación Jiménez Díaz (Madrid, Spain) on 19 December 2023 (minutes no. 22/23) for the project titled "Study of the mechanisms involved in the genesis and evolution of asthma (MEGA Project)" (PI18/01016). The present work constitutes a secondary analysis of data collected within the MEGA Project, conducted under the same approved protocol and ethical framework. The approved protocol version is Version 1, April 2023, and the informed consent version is December 2023. The study was conducted in full compliance with the Declaration of Helsinki, ICH-GCP guidelines, the General Data Protection Regulation (GDPR), and the Spanish Organic Law 3/2018 on Data Protection and Digital Rights (LOPDGDD). Written informed consent was obtained from all participants prior to their inclusion in the study. For participants who were minors, consent was obtained from their parents or legal guardians, and assent was obtained from the minors themselves, as appropriate to their age and level of understanding. All data collected were pseudonymized and securely stored to ensure confidentiality and data integrity.

*Limitations and Potential Biases*

While the sample size of our study, comprising 67 patients distributed across several biological treatments, may limit the statistical power of subgroup analyses, we believe it still provides valuable insights into real-world patterns of biological therapy in severe asthma. Some subgroups, such as those treated with tezepelumab, included a small number of patients, which should be considered when interpreting specific findings. As a single-center study conducted in a referral hospital, a certain selection bias toward more severe or complex cases cannot be excluded; however, the diversity of the patient population enhances the relevance of the observations. Moreover, although the ambispective design (retrospective and prospective) could introduce minor variability in data collection procedures over time, standardized protocols were used whenever possible to ensure data consistency. Overall, we consider our results robust and exploratory, emphasizing the need for larger, multicenter prospective studies to further validate these findings.

RESULTS

A total of 70 biologic treatments were prescribed to patients diagnosed with severe asthma who met the inclusion criteria. However, 3 patients treated with reslizumab were excluded from the analysis because the number was too small and not comparable with the other groups. Therefore, the study population included 67 patients, of whom 22 received omalizumab, 12 benralizumab, 12 mepolizumab, 15 dupilumab and 6 tezepelumab.

The baseline characteristics of all patients and grouped according to the biological agent administered are shown in Table 1 and 2. A total of 64% of the patients were women, with a mean age of 47.4 years old, and the majority were non-smokers (75%). Some of them (34.85%) were overweight (BMI between 25-30), while 28.79% had obesity (BMI > 30). The mean age of symptom onset was 25.3 years, with a mean age at diagnosis of 37.3 years. A mean diagnostic delay of 12.1 years was observed, defined as the time elapsed between the onset of asthma symptoms and the formal diagnosis of asthma by a specialist.

Among the most frequent comorbidities, rhinitis (97%) was found, followed by atopy, defined as described in the Methods - process section (82.1) and nasal polyposis (38.9%) and AERD (32.8%). Although in lower percentages, hyperventilation (7.5%), COPD (7.5%), and ABPA (2.9%) were observed. It was interesting to note that 31.3% of the sample had been under antidepressant treatment for at least 12 months or had active follow-up in mental health clinics. As for treatment, fluticasone was the molecule chosen to treat the majority of patients (65%), followed by mometasone (19.4%), beclomethasone (10.4%) and budesonide (4.5%). More than half of the patients (56.7%) **were on montelukast** as part of their maintenance therapy. Prior to biologic prescription, 19.4% (13) had an allergic T2 phenotype (positive skin prick test and/or specific IgE), 18% had an eosinophilic T2 phenotype (defined by elevated blood eosinophil counts, regardless of allergen sensitization status), and 62.6% (42) had a mixed phenotype defined by overlapping features of both allergic and eosinophilic T2 inflammation.

For the overall analysis, patients who had a previous biologic were excluded. A mean FEV1 of 72.5%±21.9% predicted, a FVC of 91.9%, a mean FVE1/FVC of 63.4±14.1% and a mean annualized exacerbation rate of 3.2±2.6 exacerbations per patient year were evident. The overall mean blood eosinophil count was 609 cells/μL, serum total IgE 685 kU/L, and FeNO 58 ppb (measured in 60 patients (89.6%). Annual cumulative oral corticosteroid dose averaged 1123.6mg±1036.7mg). About one third of all patients achieved global clinical remission (27.3%) and 12.7% reached complete remission, according to the Menzies-Gow criteria (10). The mean ACT score was 12.4, while the **nasal symptom VAS** (0–10 scale) was very low (1.3) and the prior SNOT-22 averaged 63.1. The VAS was used as a simpler retrospective tool for nasal symptom assessment in patients for whom the SNOT-22 was not available. All patients with CRSwNP underwent SNOT-22 questionnaires or VAS scale pre-treatment and at 12 months with clear improvement (SNOT-22 63.1 to 25 on average) (VAS: 1.3 to 7.2 on average).

In the exploratory comparison of baseline data among the five biologic agents, some descriptive differences were observed (Table 2). Forty patients were bio naïve and 30 (42.8%) changed therapy. It was shown that there were significant differences in the mean ages of the patients according to the biologic used (p=0.0018) with those receiving Omalizumab being the youngest (mean 38 years). No differences were found between comorbidities. The lowest FEV1 predicted basal was found in patients treated with Tezepelumab by 56.5%±17.1% with respect to the rest of the groups (p=0.080). Like Tezepelumab, it was the biologic with which the lowest FVC pred was associated (82.2%±13.2% (p=0.022). There was no difference with the ratio (p=0.084). In patients dependent on oral corticosteroids, Dupilumab presented at 4-6 months the highest mean EXACTO (7.50±2.74), while mepolizumab presented the lowest (5.60±3.58) without reaching statistical significance. However, in those who were not corticosteroid dependent Mepolizumab achieved the highest response rate at the first visit (6.00±1.10). On the other hand, Omalizumab achieved the highest FEOS score (85.8±15) although no significant differences were found between groups.

Patients were classified according to their response to biologics, based on their values in the FEOS scales (low or partial (0-89), good (90-99), very good (100)) and EXACTO (received corticosteroids: partial (0-6), good (7-9), very good (10) or no corticosteroids: partial (0-4), good (5-6), very good (7)). Sex was associated with treatment response, with females showing a higher proportion of good or very good responses compared to males, who have a higher proportion of partial responses (p=0.001). It appears that the presence of polyposis is associated with a better response to treatment (more patients with very good responses), while the absence of polyposis is more associated with partial responses (p=0.027). Although no statistically significant differences were found, a non-significant trend toward differences in treatment response at 12 months was observed in patients with AERD (p = 0.075). Peripheral blood eosinophil levels were significantly higher in patients with good and very good response compared to those with no response both at 6 (p = 0.033) and 12 months (p=0.017). Patients with a higher number of exacerbations in the previous year showed a higher proportion of good or very good responses (p = 0.036).

Clinical response after 12 months of treatment with biologics was evaluated in 55 patients, since 12 patients (Omalizumab n=1, benralizumab, n=2; mepolizumab, n=5; Dupilumab n=4) were excluded because they did not reach a treatment duration of at least 12 months (all of them with switch to another biologic due to side effects or lack of response. Overall, 27.3% of patients achieved clinical remission (15/55), while 12.7% achieved complete remission (7/55). Proportions are reported as absolute numbers and percentages. Although remission rates varied numerically across biologic therapies, no statistically significant differences were observed.

To further explore factors associated with treatment response, univariable logistic regression analyses were performed. Univariable logistic regression analyses were performed to explore clinical and inflammatory factors associated with clinical remission at 12 months (Table 3). Higher peripheral blood eosinophil counts were associated with clinical remission, whereas female sex and the presence of chronic rhinosinusitis with nasal polyps did not reach statistical significance in these models. No multivariable models could be constructed due to sample size limitations.

DISCUSSION

The present study describes the general characteristics of a cohort of patients with severe asthma seen at the allergy clinic of the Hospital Universitario de A Coruña, which allows us to contextualize the clinical profile of these patients and lay the groundwork for future studies to evaluate clinical and inflammatory factors **associated with response** to biologic therapies. Our findings suggest that female sex, comorbidities such as sinus polyposis and elevated peripheral eosinophils, and a high rate of previous exacerbations were associated with a better response to biologic therapies. Differences between associations observed in descriptive analyses and those identified in logistic regression models likely reflect the use of different outcome definitions and analytical approaches, rather than inconsistency in the findings.

On the other hand, although biologics improved asthma control in most patients, no statistically significant differences were found between biologics in terms of clinical remission, complete remission or inflammatory biomarkers as in the Valverde et al study [7]. This finding coincides with the study by Basagaña et al. [16], where complete response was associated with fewer exacerbations, but without marked differences between biologics. In our sample, 42.86% of patients had received prior biologic treatment, compared with 11.1% of switchers in the Basagaña et al. cohort [16]. This highlights the importance of assessing whether clinical response is modified in patients with prior exposure to biologics, as previous studies suggest that treatment changes may impact long-term outcomes

In general terms, our study population shows similarities with other cohorts described in recent literature. Studies such as Menzies-Gow et al [15] and Wechsler et al [17] have identified that patients with severe asthma present a high prevalence of comorbidities such as chronic rhinosinusitis with nasal polyposis and obesity, which coincides with our findings [18]. Furthermore, the distribution of inflammatory phenotypes in our sample follows the pattern observed in international studies, with predominance of eosinophilic asthma in patients with frequent exacerbations and allergic asthma in those with earlier onset of the disease Humbert et al [19,20]. Similar to the literature, patients treated with omalizumab were significantly younger, with longer disease duration, higher percentages of atopy and higher total IgE values, although it differs in a lower ACT score and higher mean FEV1 found by Bagasaña et al [16] among their patients.

On the other hand, the rate of exacerbations and the use of systemic corticosteroids in our cohort reinforce the need to optimize the therapeutic management of these patients. In comparison with the data reported in the CHRONICLE study [21], our patients have a similar frequency of exacerbations, reflecting the persistent disease burden despite standard treatment. Notably, despite the use of biologics, a significant proportion of patients continue to experience more than one or two exacerbations per year. This raises the question of whether biologic therapy is being introduced too late in the disease course, after years of uncontrolled inflammation and airway remodeling. Our results highlight a mean diagnostic delay of 12.1 years. Longer diagnostic delays may reflect prolonged periods of uncontrolled disease prior to biologic initiation; however, this hypothesis requires confirmation in prospective studies. Evidence from early-treatment studies suggests that initiating biologic therapy at an earlier stage could help mitigate airway damage and improve long-term disease control [8,22].

Regarding the use of biologic drugs, although this analysis did not focus on the response to these treatments, our data provide valuable information for future studies. The detailed characterization of biomarkers such as peripheral eosinophilia and serum IgE levels will allow further studies to evaluate which factors are associated with a better response to omalizumab, mepolizumab, benralizumab or dupilumab, in line with what has been reported so far [3,16,23].

Among the limitations of the study, it is recognized that the sample size is still limited, which could influence the generalizability of the findings. Additionally, our results highlight an important consideration regarding the current approach to selecting biologic therapy. Decision algorithms typically classify patients based on biomarkers such as skin prick test results, blood eosinophil counts, and FeNO levels [1,9,24,25]. However, our findings suggest that other clinical factors, such as the presence of nasal polyposis or AERD, may show a stronger association with treatment response than a single biomarker considered in isolation. These aspects are not always incorporated into current decision-making frameworks, yet they seem to play a crucial role in real-world practice. This raises the question of whether treatment algorithms should evolve to integrate a more comprehensive assessment of patient characteristics beyond standard biomarkers, potentially leading to a more personalized and effective approach to biologic therapy in severe asthma. Additionally, due to the exploratory nature of the study and the multiple comparisons performed across biologic subgroups and clinical variables, the potential for type I error cannot be completely excluded.

In conclusion, this real-world study in a cohort of patients with severe asthma has identified that female sex, comorbidities such as nasal polyposis, elevated peripheral eosinophils, and a high rate of previous exacerbations may be key clinical factors associated with response to biologic therapies. These findings emphasize the need to move beyond a strict biomarker-based approach and incorporate a broader clinical perspective when selecting treatment. Factors such as nasal polyposis and AERD, which are not currently part of standard decision algorithms, may play a critical role in understanding variability in therapeutic outcomes. This real-world study suggests that beyond traditional biomarkers, clinical factors such as nasal polyposis and AERD are associated with response to biologic therapies in severe asthma. These findings support the need for a broader clinical assessment to better understand response variability and to move toward a more personalized management approach.

*DISCLOSURE STATEMENT*

RLR reports receiving payments for lectures and educational events form AstraZeneca and Leti Pharma

JAC reports receiving payments for lectures and educational events form AstraZeneca and GSK.

IMF declare no conflicts of interest.

MJR reports receiving payments for lectures and educational events form A-Z, Chiesi, Gsk, Menarini, Gebro, Leti pharma

*FUNDING*

This work has been supported with the collaboration of the “Programa de la Fundación AstraZeneca Premios Jóvenes Investigadores”.

*CONTRIBUTIONS*

Raquel López.Rodríguez: Conceptualization, methodology, patient recruitment, data collection and analysis, writing—original draft, review and editing

Manuel Jorge Rial: Conceptualization, methodology, patient recruitment, data collection and analysis, writing—original draft, review and editing.

Ignacio Mahíllo-Fernández: Statistical analysis, data interpretation, writing—review and editing.

Jose Antonio Cañas: Immunological expertise, data interpretation, writing—review and editing.

All authors: Reviewed and approved the final version of the manuscript and agree to be accountable for all aspects of the work.

*DATA AVAILABILITY STATEMENT*

The data that support the findings of this study are available from the corresponding author upon reasonable request. Due to the sensitive nature of patient information, data sharing is restricted to comply with the General Data Protection Regulation (GDPR) and national data protection laws. Requests for data access will be considered on a case-by-case basis, subject to ethical approval and compliance with applicable regulation

*References:*

1. *Wechsler ME, Munitz A, Ackerman SJ, Drake MG, Castro M. Biologic therapies for severe asthma. N Engl J Med. 2023;389:1340-1353*
2. *Mukherjee M, Aleman Paramo F, Kjarsgaard M, Salter B, Nair G, LaVigne N, et al. Predictors of response to biologics in type 2 high severe asthma. J Allergy Clin Immunol Pract. 2023;11(1):145-155.*
3. *López-Rodríguez R, Bobolea I, Melero C, Rial MJ. Editorial: A multidisciplinary approach to treatment of severe chronic airway disease (CAD): focus on biomarkers. Front Allergy. 2024 Feb 2;5:1357886*
4. *Rial MJ, Betancor D, Cañas JA, Olaguibel JM, Rodrigo-Muñoz JM, Alvarez-Puebla MJ,et al. Stability of Asthma Phenotypes in the Spanish Cohort of the MEGA Project. J Investig Allergol Clin Immunol. 2025 Feb 18;35(1):53-55*
5. *Upham JW, Chung LP, Reddel HK, Marks GB, Rimmer J, Hallstrand TS, et al. Upham JW, et al. Defining a severe-asthma super-responder: findings from a Delphi process. J Allergy Clin Immunol Pract. 2021;9(7):2798-2805.*
6. *Demolder F, Vanderhelst E, Verbanck S, Schuermans D, Brusselle G, Dupont L, et al. Real-life response to biologics in severe asthma with nasal polyposis: insights from the Belgian Severe Asthma Registry. Lung. 2024;202(4):441-448. doi:10.1007/s00408-024-00715-0*
7. *Valverde-Monge M, Sánchez-Carrasco P, Betancor D, Barroso B, Rodrigo-Muñoz JM, Mahillo-Fernández I, et al. Comparison of Long-term Response and Remission to Omalizumab and Anti-IL-5/IL-5R Using Different Criteria in a Real-life Cohort of Severe Asthma Patients. Arch Bronconeumol. 2024 Jan;60(1):23-32*
8. *Pérez-de Llano L, Bonilla MG, Luzon E, Ramos-Lima F, Miralles JC. What we know to date about early treatment with biologics in severe asthma: a scoping review about the use of biologics in severe asthma and possible parallels with early rheumatoid arthritis. Open Respir Arch. 2025;7(2):100418. doi:10.1016/j.opresp.2025.100418*
9. *Spanish Guide for Asthma Management, 2024 update (GEMA 5.4). SEPAR; 2024. Available from:* [https://www.gemasma.com](https://www.gemasma.com/) (last accesed March-18-2025)
10. *Jia CE, Zhang HP, Lv Y, et  al. The asthma control test and asthma control questionnaire for assessing asthma control: systematic review and meta-analysis. J Allergy Clin Immunol. 2013;131(3):695–03.*
11. *Alvarez-Gutiérrez FJ, Blanco-Aparicio M, Casas-Maldonado F, et al. EXACTO scale: multidimensional tool for assessing the response to treatment with monoclonal antibodies in severe uncontrolled asthma. Open Respiratory Archives. 2022;4:100192*
12. *Perez de Llano L, et al. Development of a tool to measure the clinical response to biologic therapy in uncontrolled severe asthma: The FEV1, exacerbations, oral corticosteroids, symptoms score. J Allergy Clin Immunol Pract. 2021;9(7):2725-31.*
13. *Hung KF, Wenzel SE, Brozek JL, et al. International ERS/ ATSguidelines on definition, evaluation and treatment of severe asthma. Eur Respir J. 2014;43(2):1–14.*
14. *Rial MJ, Domínguez-Ortega J. Inflammatory Remission in T2 Severe Asthma. Front Allergy. 2022 May 30;3:923083*
15. *Menzies-Gow A, Bafadhel M, Busse WW, Casale TB, Kocks JWH, Pavord ID, et al. An expert consensus framework for asthma remission as a treatment goal. J Allergy Clin Immunol. 2020;145(3):757-65.*
16. *Basagaña M, Martínez-Rivera C, Padró C, Garcia-Olivé I, Martínez-Colls M, Navarro L, et al. Clinical characteristics of complete responders versus non-complete responders to omalizumab, benralizumab and mepolizumab in patients with severe asthma: a long-term retrospective analysis. Ann Med. 2024 Dec;56(1):2317356.*
17. *Wechsler ME, Scelo G, Larenas-Linnemann DES, Torres-Duque CA, Maspero J, Tran TN, et al. Association Between T2-related Comorbidities and Effectiveness of Biologics in Severe Asthma. Am J Respir Crit Care Med. 2024 Feb 1;209(3):262-272.*
18. *Barroso B, Valverde-Monge M, Alobid I, Olaguibel JM, Rial MJ, Quirce S, et al. Improvement in Olfaction in Patients With CRSwNP and Severe Asthma Taking Anti-IgE and Anti-IL-5 Biologics: A Real-Life Study. J Investig Allergol Clin Immunol. 2023 Feb 17;33(1):37-44*
19. *Humbert M, Busse W, Hanania NA, Lowe PJ, Canvin J, Erpenbeck VJ, et al. Phenotypes and endotypes of adult asthma: moving toward precision medicine. J Allergy Clin Immunol. 2019 Jul;144(1):1-12.*
20. *Matabuena M, Salgado FJ, Nieto-Fontarigo JJ, Álvarez-Puebla MJ, Arismendi E, Barranco P, et al. Identification of Asthma Phenotypes in the Spanish MEGA Cohort Study Using Cluster Analysis. Arch Bronconeumol. 2023 Apr;59(4):223-31*
21. *Lugogo N, Judson E, Haight E, Trudo F, Chipps BE, Trevor J, et al. Severe asthma exacerbation rates are increased among female, Black, Hispanic, and younger adult patients: results from the US CHRONICLE study. J Asthma. 2022;59(12):2495-08. 20*
22. *Betancor D, Bautista S, López-Rodríguez R, Valverde-Monge M, Fernández-Nieto M, Rial MJ. Four-month real-life response to Tezepelumab in patients with multi-failure to other biologics. Allergol Immunopathol (Madr). 2024 Nov 1;52(6):76-78*
23. *Efficacy of Biologics in Patients with Allergic Severe Asthma, Overall and by Blood Eosinophil Count: A Literature Review. Adv Ther. 2023;40(10):4535-50.*
24. *Pavord ID, Bafadhel M. Exhaled nitric oxide and blood eosinophilia: independent markers of preventable risk. J Allergy Clin Immunol. 2013 Oct;132(4):828-9*
25. *Betancor D, Olaguibel JM, Rodrigo-Muñoz JM, Arismendi E, Barranco P, Barroso B, et al. How reliably can algorithms identify eosinophilic asthma phenotypes using non-invasive biomarkers? Clin Transl Allergy. 2022 Aug 20;12(8):e12182*
