# Supplementary material for: Beyond biomarkers: the role of clinical factors associated with biologic therapy response in severe asthma
Source: Ann Med. 2026 Feb 11;58(1):2627026. doi: 10.1080/07853890.2026.2627026 (PMC12895875; doi:10.1080/07853890.2026.2627026)
Supplement: Figure 1 legend.docx [file IANN_A_2627026_SM6895.docx]

**Figure 1.** **Flow diagram showing patient selection and exclusion process for the final analysis of severe asthma patients treated with biologics**
